# Inclusion and Functionalization of Polymers with Cyclodextrins: Current Applications and Future Prospects

**DOI:** 10.3390/molecules190914066

**Published:** 2014-09-09

**Authors:** Christian Folch-Cano, Mehrdad Yazdani-Pedram, Claudio Olea-Azar

**Affiliations:** 1 Departamento de Química Orgánica y Fisicoquímica, Universidad de Chile, Sergio Livingstone 1007, P.O. Box 233, Independencia, Santiago 8380492, Chile; 2 Departamento de Química Inorgánica y Analítica, Facultad de Ciencias Químicas y Farmacéuticas, Universidad de Chile, Casilla 233, Santiago 8380492, Chile

**Keywords:** cyclodextrins, inclusion complexes, molecularly imprinted polymers, polymers, ternary complexes

## Abstract

The numerous hydroxyl groups available in cyclodextrins are active sites that can form different types of linkages. They can be crosslinked with one another, or they can be derivatized to produce monomers that can form linear or branched networks. Moreover, they can form inclusion complexes with polymers and different substrates, modifying their physicochemical properties. This review shows the different applications using polymers with cyclodextrins, either by forming inclusion complexes, ternary complexes, networks, or molecularly imprinted polymers (MIPs). On one hand, the use of cyclodextrins enhances the properties of each polymer, and on the other the use of polymers decreases the amount of cyclodextrins required in different formulations. Both cyclodextrins and polymers contribute synergistically in several applications such as pharmacological, nutritional, environmental, and other industrial fields. The use of polymers based on cyclodextrins is a low cost easy to use potential tool with great future prospects.

## 1. Introduction

Cyclodextrins (CDs) are cyclic oligosaccharides composed of glucose units connected by α-1,4-glycosidic linkages with numerous available hydroxyl groups that are active sites for forming different types of derivatives and linkages. They are truncated-cone-shaped, with a relatively hydrophobic cavity and an external hydrophilic surface, capable of forming inclusion complexes (ICs) with various molecules, by incorporating them into the cavity [1,2,3]. The formation of ICs can modify the physical, chemical, and biological properties of the guest molecule, expanding its application field. The ability of a CD to form an IC with a guest molecule is a function of steric as well as thermodynamic factors. The driving forces for complex formation are attributed to the removal of water molecules from the hydrophobic cavity and the formation of Van der Waals, hydrophobic, and hydrogen bond interactions [4,5]. CDs have been recommended for applications in food processing and as food additives with various aims, like protecting lipophilic food components that are oxidizable and degradable by light or heat, solubilizing vitamins and dyes, encapsulating flavours, fragances, and essential oils against unwanted changes, suppressing unpleasant odors or tastes; and achieving controlled release of certain food and drug constituents [6,7,8,9].

**Figure 1:**
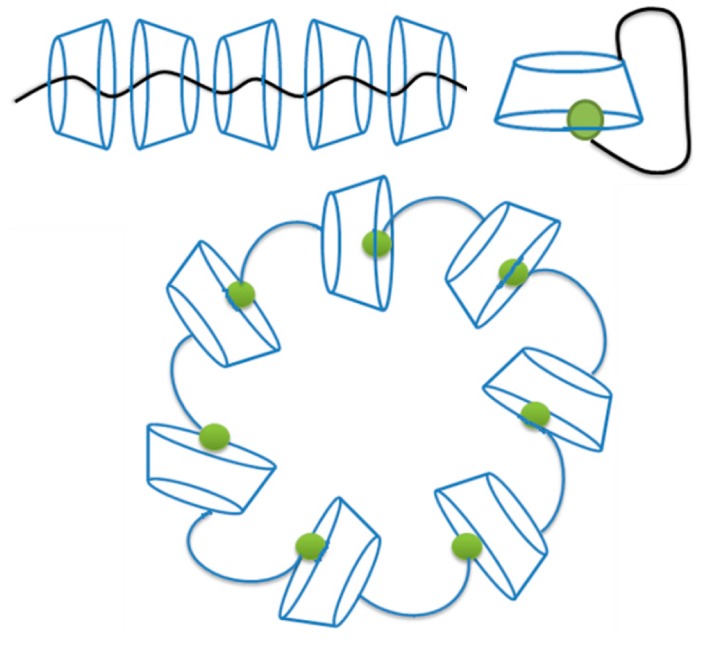
Schematic representation of linear complexation of a polymer chain by CDs and CDs derived with monomeric fractions forming a ring type arrangement.

CDs have been found to form ICs not only with low molecular weight compounds. New molecular structures and functions have been obtained by the inclusion of polymer chains in the cavities of CDs [10]. Figure 1 shows a schematic representation of some IC between them. Solid state NMR spectroscopy is an efficient technique to study the conformations and molecular motions of polymer inclusion complexes. Lu *et al.* [11] studied the conformations and dynamics of series of crystalline polymers. The ICs obtained showed single chains for urea and perhydrotriphenylene with α-CD and double chains for poly(ethylene oxide) and poly(ϵ-caprolactone) with γ-CD channels, like the double stranded occupancy of poly(ethylene glycol)-γ-CD IC exposed by Harada *et al.* [12]

In this review we will show the different applications made possible by the use of polymers with cyclodextrins, either by forming inclusion complexes, ternary complexes, networks and molecularly imprinted polymers (MIPs). On one hand, the use of cyclodextrins enhances the properties of each polymer, and on the other the use of polymers decreases the amount of cyclodextrins necessary in different formulations. Both cyclodextrins and polymers contribute synergistically in several applications such as in the pharmacological, nutritional, environmental and general industrial fields, making the use of polymers based on cyclodextrins a low cost, easy to use potential tool with great future prospects.

## 2. Polymers as Ternary Complex Constituents

The encapsulating properties of CDs can be improved by forming ternary complexes. The ternary complexes are supramolecular systems composed of three distinct molecular entities. From this point of view, this ternary system would be represented by an auxiliary substance which, in conjunction with CD, can improve the physicochemical, chemical and bioavailability properties of a guest molecule. Depending on the case, the use of a third component improves encapsulation or release and reduces the amount of CD needed to obtain the benefits mentioned above. In this way can optimize the cost, toxicity, and bulk formulation of the desired product.

Polymers are the most widely used ternary components in drug/CD systems [9]. Faucci *et al.* [13] studied the effect of water soluble polymers like sodium carboxymethylcellulose, hydroxylpropyl-methylcellulose, polyvinylpyrrolidone K30, and polyethylene glycol 6000 on naproxen solubility improvement. The combined use of polymer and CD was always clearly more effective in enhancing the aqueous solubility of naproxen compared to the corresponding drug/polymer or drug/CD binary systems. The solubilization enhancement was not simply additive, but synergistic. Their addition leads to decreased drug cristallinity and synergetic effects on the solubilizing action of CDs. For example, in polyvinylpyrrolidone/CD/drug systems, an increase in the solubility of the drugs was experienced under the polymer's effect. However, the increase in the solubility was not greater than for other polymers like hydroxypropylmethylcellulose or plasdone [1].

## 3. Networks of Polymers and Cyclodextrins

The first approaches to the potential applications of cyclodextrin polymers (soluble and insoluble) in the pharmaceutical industry were reviewed by Fenivesy [14] and Szeman *et al.* [15] These authors studied the complex-forming ability of a water-soluble β-CD epichlorohydrin, a polymer that formed quite soluble inclusion compounds with some drugs. Renard *et al.* [16] established that the molar weight of these water soluble polymers depends on initial features like initial ratio (epichlorohydrin/β-CD), NaOH concentration, and reaction time. They indicated that low concentrations of NaOH promote substitution on the two sides of the CD cavity, while high NaOH concentrations result in substitution mainly on one side of the cavity.

**Figure 2:**
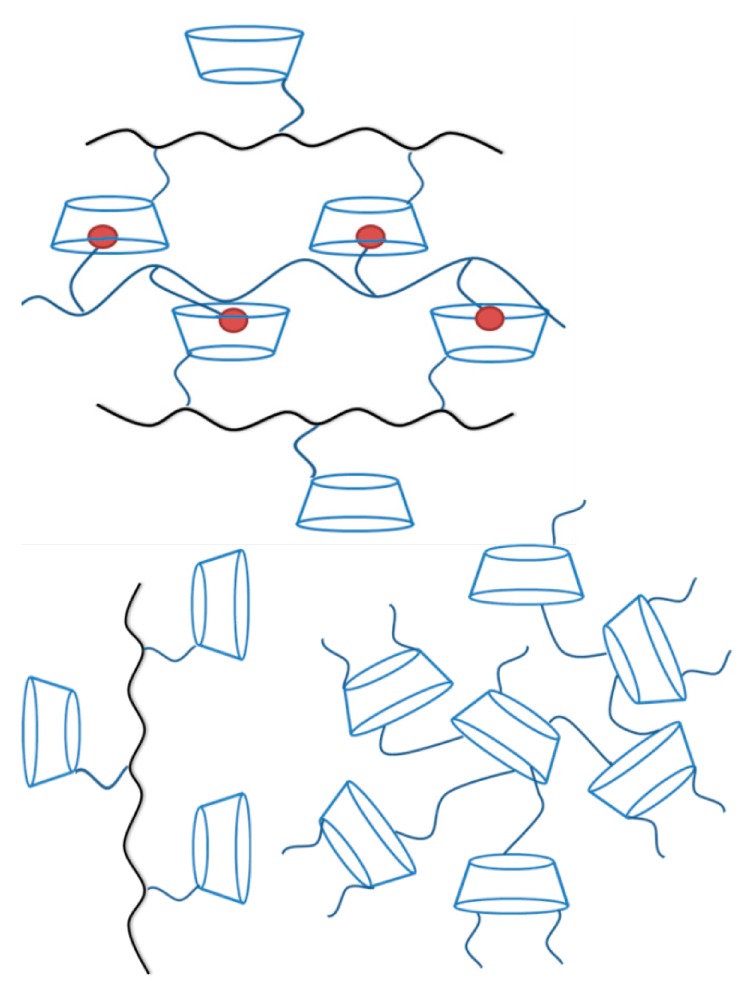
Scheme of different polymer-based CD networks.

There are several procedures for the conversion of β-CD into derivatives capable of interacting with compounds from aqueous solutions, and the methods can be classified in two ways. The first method involves the direct modification of β-CD by cross-linking, which consists in the bonding of the molecules to each other, by concatenation through a coupling agent, to form an insoluble three-dimensional network, as schematized in Figure 2. The properties can be adjusted according to the type of CD, the cross-linking spacer, or the molar ratio between the two, getting materials with different characteristics in terms of density, surface area, pore structure, chemical properties, and sorption behavior.

Covalent polymer networks containing cyclodextrins are of great interest because their croslinked macromolecular structure has a cooperative action between the cyclodextrin cavities, or between the cavities and the polymeric network, in the complexation and adsorption of water soluble components. From this perspective, CD polymers were prepared by solubilization of citric acid as crosslinking agent and sodium hypophosphite as catalyst [17,18], and they were used to improve the complexation of some drugs. These polymers with an anionic character present low toxicity and an environmentally friendly composition.

The second method is based on the covalent binding of β-CD molecules to a pre-existing insoluble matrix such as highly cross-linked synthetic polymers. This kind of CD immobilization on insoluble supports can be regarded as an appropriate way to get insoluble CD materials with specific properties like functional delivery additives or to act as sensors or filters [19]. The hydroxyl groups of CDs have been successfully functionalized with vinyl groups by reacting with acryloyl chloride to produce an ester. Homo- and copolymerization of this modified CD monomer are being explored using free radical initiators. Comonomers such as *t*-butyl acrylate, methyl methacrylate and styrene are being used, since their side groups confer solubility in common solvents. The CD content of the copolymer can be controlled by varying the comonomer content [20]. Munteanu *et al.* [21] synthesized such polymerizable CD monomers via a “click chemistry” route leading to a ring-like supramolecular structure. Copper(I)-catalyzed Huisgen type 1,3-dipolar cycloadition of azides and alkynes extended its area of application. By applying a click reaction, CD monomethacrylate can be obtained in high yields; purification by chromatographic methods is not required, and installation and removal of protecting groups is avoided. The triazol linker is relatively stable toward cleavage, oxidation, or reduction. Click reactions as a possibility to obtain polymer chains with covalently attached CDs and examples of the application of such polymers in drug delivery have been reported [22].

Doyagües *et al.* [23] reported the synthesis and aqueous catalytic evaluation of a linear copolymer bearing both pendant proline and permethylated β-CD groups. They showed the synthesis of the protected hydroxyproline methacrylate and permethylated 6-azido-6-deoxy-β-cyclodextrin used as a precursor of styrenic compounds. The copolymer was designed on the basis that the presence of the hydrophobic cavity of the β-CD could bring aromatic substrates into close proximity of the surrounding catalytic proline residues through host−guest interactions. The compound is water-soluble and catalyzes aldol reactions in this medium without the need for any extra organic solvent. The copolymeric catalyst showed a pH-dependent behavior. At pH 7.0 the copolymer is found as extended single chains with negative charge, catalyzing the reaction in a fast and nonstereoselective mode. On the other hand, at pH 3.8 it is forming charge complexes, multichain hydrophobic nanoaggregates that have probably been stabilized by the permethylated-β-CD presence.

Based on the background outlined above, the click reaction allows synthesizing monomers of some polymers functionalized with permethylathed-β-CD. Moreover, this monomer can be copolymerized by a bottom-up route. Moers *et al.* [24] synthesized monoadamantyl-functional hyperbranched and linear–hyperbranched polyglycerols by a self-assembly process. Polymers with molecular weights ranging from 2700 to 12,100 g/mol were obtained. The association constant was found to drop with increasing molecular weight of the hyperbranched polyglycerol, but it levels off to a final value already at moderate molecular weights of the hyperbranched block. Additionally, the presence of the polyethylene glycol spacer between the adamantyl group and the hyperbranched polyglycerol resulted in significantly increased association constants, demonstrating the steric effect of branching.

Another alternative is the simple and well-known chemical crosslinking reaction using epichlorohydrin as cross-linker. This cross-linker contains two reactive functional groups that can form bonds with CD molecules and/or itself. The resulting polymers are mixtures containing CD units joined by repeating glyceryl linkers. A number of CDs are interconnected and a three-dimensional (co)polymer network is formed. These (co)polymers are called gels or hydrogels due to their highly hydrophilic nature. A large number of papers have been published on the synthesis and use of these gels, and they have been reviewed by Crini *et al.* [19].

The epichlorohydrin-β-CD polymers were used by Zhao *et al.* [25] to prepare a novel β-CD polymer/tungsten carbide (CroCD-TuC), comparing it with Sephadex G15 beads in terms of their adsorbing capacity for rutin [26]. They concluded that its adsorption isotherms (Langmuir adsorption equation) and adsorption capacity are gradually reduced as the solution's temperature and pH rise, but it increases with increasing solvent polarity. Thermodynamic studies revealed that the formation of the rutin-β-CD complex CroCD-TuC skeleton showed significant contribution to the adsorption compared to Sephadex. In a modified interfacial polymerization, nylon-6,10 containing various amounts of covalently bonded dimethyl-βCD has been obtained by Tonelli [27].

These dimethyl-β-CD-nylon-6,10 polymers showed thermal degradation characteristics very similar to pure nylon-6,10, and they are more readily dyed at higher dye levels than pure nylon-6,10 due to the β-CD effect.

## 4. Molecularly Imprinted Polymers (MIPs) with Cyclodextrins

**Figure 3:**
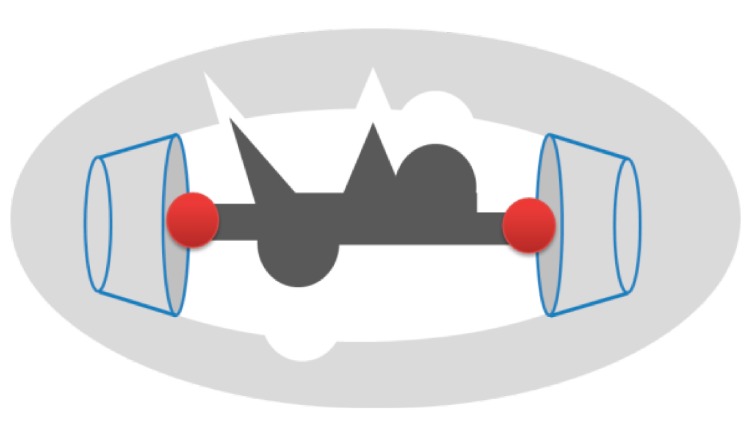
Schematic representation of a hydrophobic template molecule in a CD-based MIP.

In order to increase the adsorption selectivity of some polymers, molecularly imprinted molecules have been designed. Their application is mainly oriented at the recognition of target molecules in biological systems such as artificial receptors, and also in analytical separation processes. In the gest-binding sites it is likely that CDs are arranged complementarily to the template in the polymer. To immobilize and determine the position of CD residues in the polymer, two or more hydrophobic molecules that can form ICs with CDs should be fixed in the template (Figure 3).

Asanuma *et al.* [28,29] synthesized acryloyl-CDs as functional vinyl monomers, and various antibiotics and oligopeptides were molecularly-imprinted to them in water. They proposed that the imprinting effect is eminent when the template involved more than two hydrophobic residues on a rigid molecular frame promoting guest selectivity including enantioselectivity.

Egawa *et al.* [30] prepared microspheres of molecular imprinted CDs in a dimethylsulfoxide/poly(dimethylsiloxane) emulsion, using cholesterol and other steroids as template molecules. Cholesterol-specific binding ability into the MIP was most remarkable and they proposed it as a novel cholesterol selective MIP.

The high selectivity of CDs based on MIP was corroborated by Guo *et al.* [31], who reported a novel approach for preparing a MIP with CD pseudo-polyrotaxanes as pseudo-supports, which are formed by self-assembling assistant recognition polymer chains with γ-CD. For these purposes, template bovine serum albumin (BSA) was first selectively assembled with modified CD pseudo-polyrotaxanes to form IC in the presence of Cu ions. The MIP was characterized by 2D-NOESY, thermogravimetric analysis (TGA), and wide-angle X-ray diffraction (WARD). Other β-CD-based MIPs were synthesized using β-CD as monomer that is cross-linked by toluene-2,4-diisocyanate with different template molecules. Kyzas *et al.* [32] prepared MIPs using Remazol Red 3BS (RR) as template molecule. The selectivity of the MIP was elucidated in a trichromatic dye solution. Its regeneration/reuse capability was dependent on pH. The CD-MIPs showed higher rebinding of dye than chitosan MIPs prepared in the study. *N*-phenyl-1-naphthylamine was also used as template molecule by Ng *et al.* [33], showing better sensing signal by increasing the binding affinity and substrate-selectivity towards the template molecule compared with the control polymer prepared in its absence. Another template used for this type of MIP was dextromethorphan, which is an important pharmacological marker drug used to identify the activity of the CYP2D6 class of p450 monooxygenases [34]. Detection and quantification were made by measuring the refractive index changes of multiple surface plasmons resulting from the binding of imprinted βCD polymer in pockets.

On the other hand, β-CD was also used as a monomer cross-linked with epichlorohydrin in the presence of creatinine, which was a template for the imprinting [35]. *N*-hydroxysuccinimide and 2-pyrrolinidone were used as creatinine analogues in the adsorption of multi-component solutions to reveal the specific recognition ability of the abovementioned MIP for the creatinine template molecule. The specific binding of the creatinine-MIP was confirmed. The adsorption resulting from the solution mixture by MIP showed superior binding effect for the target molecule, creatinine, rather than creatine, *N*-hydroxysuccinimide, and 2-pyrrolinidone.

A novel photonic MIP was designed for amino acid sensing using maleic anhydride-modified β-CD and acrylic acid as functional monomers [36]. This β-CD-based MIP could undergo a swelling change from 590 nm to 704 nm and still retain the molecular imprinting recognition ability during the sensing of l-phenylalanine. The authors proposed it as a sensor to detect l-phenylalanine in compound amino acid injection samples due to this high specificity with respect to d-phenylalanine, l-tyrosine, and l-tryptophan.

## 5. Current Applications of Polymers and CD Networks

Because CDs are nontoxic, biodegradable and bioabsorbable, they may be used in polymeric networks for varied purposes oriented mainly at applications like food, environmental for removing organic pollutants and heavy metal ions from water, and pharmacological, as listed in Table 1.

**Table 1:** Reported applications of some CD-polymeric networks.

Polymer	Application		Ref.
β- and γ-CDs linked with chitosan	Bitter-masking of caffeine solutions and bitter natural extracts		[37]
β-CD with one of the following crosslinking agents: epichlorohydrin, diphenyl carbonate, or hexamethylene diisocyanate and propargyl-β-CD with 1,3-bis(azidomethyl) benzene	Debittering agents by adsorption of narangin, limonin and caffeine		[38]
β-CD and epichlorohydrin	Environmental purposes: detoxification of wastewater, color removal, concentration and purification of solutions		[19]
	Delivery of *Lavandula angustifolia* essential oil used as ambient odors		[39]
	Retention of aroma compounds of *Mentha piperita* esential oil		[40]
	Adsorption of pesticides from water		[41]
	Removal of Cu^2+^ from aqueous solutionss		[42]
β-CD and anionic and cationic acrylamide	Enhancement of oil recovery		[22]
Chemically cross-linked and grafted cyclodextrins	Drug release from hydrogels		[28]
α-, β- and γ-CDs functionalized with acrylic groups	Aqueous nanogels		[43]
β-CD polymer/tungsten carbide	Adsorption of rutin		[26]
β-CD, acrylic acid, *N*,*N'*-methylenebisacrylamide		Hydrogel for the removal of heavy metal ions	[44]
Monomethacrylated β-CD copolymerized with *N*-isopropylacrylamide		Drug delivery	[21]
Oligosaccharide γ-CD with dibasic acid dichlorides		Adsorption of polychlorobiphenyl contaminants in oil	[45]
β-CD-citric acid		Drug delivery of ciprofloxacin (an antibiotic) and prednisolone (an anti-inflammatory drug)	[46]
β-CD-dextran polymers		Drug delivery	[47]
HPγ-CD polymer and sulfobutylether-β-CD with epichlorohydrin		Adsorption of ionizable oxytetracycline antibiotics	[48]
Phosphorous-containing β-CD polymers		1-adamantyl acetic acid, or with divalent cations, such as Ca^2+^	[49]
CD crosslinked with 4,4'-methylenebis(phenyl isocyanate)		Removal of patulin from apple juice	[50]
Glycidyl methacrylate alone or in combination with β-CD		Incorporation of insecticide in cotton textiles	[51]
α-CD-polymer gels with various poly(ethylene oxide) (PEO)-based copolymers		Delivery of vancomycin for the treatment of bone infections	[52]
From β-CD and sulfobutylether-β-CD with epichlorohydrin		Solubilization of repaglinide (hypoglycemic agent)	[53]
β-CD-ionic liquid polymer with 1-benzylimidazole and crosslinked using toluenediisocyanate		Sorption capacity and high removal towards phenols and arsenic(V)	[54]
Carboxymethyl-hydroxypropyl-β-CD polymer-modified Fe_3_O_4_ magnetic particles (CM-HP-β-CDCP-MNPs)		Magnetic solid phase extraction of rutin	[55]
Crosslinked polyvinyl alcohol/glutaraldehyde (PVA/GA) membranes with β-CD		Textile liquid waste processing	[56]
PCCA thin film modified with β-CD polymer		Capping cavity for the selective detection of paraoxon-ethyl and parathion-ethyl chemical agents	[57]

Cyclodextrin-based polymers have been shown to be efficient drug delivery materials; βCD-citric acid *in-situ* polymerization was used to functionalize two kinds of porous silicon (nanoporous and macroporous) that were used as matrices for drug release studies either in water or phosphate buffer solutions using ciprofloxacin and prednisolone as model drugs. Both cyclodextrin-based polymer composites showed better release control for drug delivery applications than non-functionalized silicon-β-CD-citric acid composite [46]. The drug delivery for eventual parenteral administration of poorly soluble drugs was also studied by using novel β-CD-dextran polymers [47]. In this study the size distribution of β-CD-dextrans and the influence of dextran backbones on the stability of the β-CD/drug complex as well as the solubilization efficiency were investigated. The stability of the β-CD/drug complex was not affected by the dextran backbone compared to native and commercially available β-CD derivatives. It was concluded that these new polymers have higher drug solubilization capacity compared to commercially available β-CDs.

Supramolecular gels showed good cytocompatibility against SAOS-2 cells and in the HET-CAM test [52]. The supramolecular gels of α-cyclodextrin-polymer with various poly(ethylene oxide) (PEO)-based water-soluble copolymers were synthesized from α-CD by crosslinking with epichlorohydrin in alkaline medium and they were able to sustain the release of vancomycin for at least 5 days at 37 °C, more efficiently than dispersions of either polymer component separately, resulting in a potential controlled delivery system for the treatment of bone infections.

Cross-linked CD-based nanosponges, from β-CD and sulfobutylether-β-CD with epichlorohydrin were synthesized [53]. The complexing properties of the polymers were investigated against repaglinide (a hypoglycemic agent, practically insoluble in water), providing higher solubilization ability. The amount of dissolved repaglinide is almost twice the amount dissolved in the presence of CD monomers. They proposed that the lower solubilization effect of SBEβ-CD-NS is probably due to steric hindrance, though its loading capacity is significantly higher.

From an environmental perspective, the spherical porous CD polymers prepared with one-fold or composite CDs as complexing agents and epichlorohydrin as cross-linking reagent were studied in terms of the relationship between adsorption potential and adsorbent-adsorbate, and their ability to adsorb and separate pesticides from water [41] was investigated by environmental adsorbent design, studying the effects of pH on adsorption and removal efficiency of ionizable organic compounds [48]. In the latter study, the authors checked the pH-dependent adsorption of the ionizable antibiotic oxytetracycline (comprising OTCH2^+^, OTCH^±^, OTC^−^, and OTC2^−^) on cyclodextrin polymers from the standpoint of molecular recognition and pH inertness. Based on these findings, they proposed HPγ-CD polymers to adsorb and remove oxytetracycline at pH 5.0 and 7.0, showing high removal efficiency and strong resistance to the interference of coexisting components.

Kawano *et al.* [45] cross-linked the renewable cyclic oligosaccharide γ-CD with dibasic acid dichlorides, as a new selective and powerful adsorbent to remove polychlorobiphenyl contaminants from oil (with >99.9% recovery efficiency) by simply washing with acetone.

The adsorption-desorption trends of epichlorohydrin cross-linked with β-CD, along with its good recyclability, establish an alternative, effective, and novel remediation technology for the removal of Cu^2+^ from aqueous solutions. The adsorption of Cu^2+^ was observed to be higher at pH 6.0 and showed a pseudo-second-order kinetic model. The maximum binding of Cu^2+^ was estimated to be 111.11 mg/g through the Langmuir isotherm model—much higher than that of existing sorption technologies [42].

From an electrochemical perspective, Zhang *et al.* [58] functionalized carbon nanomaterials modified with β-CD polymers that been bonded by non-covalent interactions. They prepared electrodes modified with the nanomaterials that exhibited high electrochemical performance with high supramolecular recognition properties.

Novel phosphorous-containing β-CD polymers with molecular weights higher than 10^4^ g·mol^−1^ were synthesized by polycondensation between the hydroxyl groups of β-CD and non-toxic sodium trimetaphosphate under “green chemistry” conditions. These were non-reticulated and soluble CDs. Strong interactions with probes such as 1-adamantyl acetic acid or with divalent cations such as Ca^2+^ were observed. This was attributed to the presence of β-CD and phosphate groups in the polymer. The phosphated compounds obtained also displayed high affinity towards hydroxyapatite, leading to a quantitative determination of the total amount of phosphate molecules fixed on hydroxyapatite [49].

Β-CD-ionic liquid polymer (CD-ILP) was first synthesized by β-CD functionalized with 1-benzylimidazole (BIM) to form monofunctionalized CD (β-CD-BIMOTs), and it was further polymerized using a toluene diisocyanate linker to form insoluble CD-ILP (β-CD-BIMOTs-TDI) [54]. The presence of ionic liquid (IL) increases pore size, while the thermogravimetric analysis result showed that the presence of IL increases the stability of the polymer. The authors showed that the β-CD-BIMOTs-TDI polymer presents enhanced sorption capacity and high removal of phenols and arsenic(V).

Carboxymethyl-hydroxypropyl-β-CD polymer-modified Fe_3_O_4_ magnetic particles (CM-HP-β-CDCP-MNPs) were prepared and applied to magnetic solid phase extraction of rutin combined with UV–visible spectrometric detection [55]. The maximum adsorption capacity was high for rutin, with short equilibrium time and the possibility of reusing the adsorbent up to ten times.

Another applied perspective shows the study of Shirasawa *et al.* [50], who used a CD-based polymer for patulin analysis in apples. An insoluble polymer composed of cyclodextrin crosslinked with 4,4'-methylenebis(phenylisocyanate) was synthesized for use in the solid phase extraction of patulin from apple juice.

In an innovative perspective, cotton textiles which act against insects such as mosquitoes were designed by the incorporation of efficient insecticides (permethrin, bioallethrin) in the macro-molecular structure of cotton fabrics modified by grafting. The grafting reaction was carried out with glycidyl methacrylate or combinated with β-CD by irradiation using fast electron beams [51]. The amount of insecticide incorporated in the grafted cotton fabrics was increased by increasing the amount of β-CD. These grafted cotton fabrics showed fast action against mosquitoes.

The ability of cyclodextrin to include a wide variety of chemicals was also exploited for dye adsorption to show the potentialities of the membranes in textile liquid waste processing [56]. Adsorption of reactive methyl orange and methylene blue dyes on crosslinked polyvinyl alcohol/glutaraldehyde (PVA/GA) membranes was carried out, and attempts to obtain hydrophilic crosslinked PVA membranes were made by adding various amounts of β-CD. The membranes were consequently studied using UV-Vis spectroscopy at wavelengths of 547, 463, and 660 nm. The authors indicated that there is no covalent bond formation between PVA and β-CD; the β-CD is completely mixed into the PVA matrix polymer. The adsorption capacity increases with increasing amounts of cyclodextrin and the change in adsorption capacity may be due to the dye structure effect, and the negative free energy indicated the spontaneous nature of adsorption.

An optical chemical sensor for the detection of organophosphate (OP) compounds using a polymerized crystalline colloidal array (PCCA) thin film composed of a close-packed colloidal array of polystyrene particles was developed [57]. The PCCA thin film was modified with β-CD polymer as a capping cavity for the selective detection of paraoxon-ethyl and parathion-ethyl chemical agents. A fast response time (10 s) and high sensitivity with detection limits of 2.0 and 3.4 ppb for the abovementioned compounds were established by the authors.

## 6. Conclusions

The encapsulating characteristics of cyclodextrins, accompanied by the ease with which they can be derivatized, allows the formation of inclusion complexes with various polymers, with the possibility of crosslinking them as well as of forming monomers, which can then be part of polymeric networks, with synergistic properties that can enhance the properties of both the cyclodextrins and the polymers, leading to the modification of the physicochemical properties of some adsorbates or guest molecules. Currently, these cyclodextrin-based polymers are used mainly in drug delivery processes, gel design, encapsulation of specific molecules in food matrices, environmental applications, and in the creation of new textile products, among others. Additionally, new applications focused on increasing the selectivity properties, oriented at biochemical processes, are currently being addressed through the design and use of molecularly imprinted polymers containing cyclodextrins. The design and use of the properties of polymers and cyclodextrins, the wide range of existing studies and their potential applications are undoubtedly the starting point of much future research.
